# Supplementary material for: Enrichment Methods for Murine Liver Non-Parenchymal Cells Differentially Affect Their Immunophenotype and Responsiveness towards Stimulation
Source: Int J Mol Sci. 2022 Jun 11;23(12):6543. doi: 10.3390/ijms23126543 (PMC9223567; doi:10.3390/ijms23126543)
Supplement: Supplementary file 1 [file ijms-23-06543-s001.zip › ijms-1757523-supplementary.pdf]

International Journal of *Molecular Sciences*

Supplementals

Enrichment Methods for Murine Liver Non-Parenchymal Cells Differentially Affect Their Immunophenotype and Responsiveness towards Stimulation

Carolina Medina-Montano [1,†], Maximiliano Luis Cacicedo [2,†], Malin Svensson [2], Maria Jose Limeres [2], Yanira Zeyn [1], Jean Emiro Chaves-Giraldo [1], Nadine Röhrig [1], Stephan Grabbe [1], Stephan Gehring [2,†] and Matthias Bros [1,*,†]

[1] Department of Dermatology, University Medical Center of the Johannes Gutenberg University Mainz, Langenbeckstraße 1, 55131 Mainz, Germany; gmedinam@uni-mainz.de (C.M.-M.); yanira.zeyn@uni-mainz.de (Y.Z.); jchavesg@students.uni-mainz.de (J.E.C.-G.); n.roehrig@uni-mainz.de (N.R.); stephan.grabbe@unimedizin-mainz.de (S.G.)

[2] Children's Hospital, University Medical Center of the Johannes Gutenberg University Mainz, Langenbeckstraße 1, 55131 Mainz, Germany; mcaciced@uni-mainz.de (M.L.C.); malin.svensson@uni-mainz.de (M.S.); mj.limeres@uni-mainz.de (M.J.L.); stephan.gehring@uni-mainz.de (S.G.)

* Correspondence: mbros@uni-mainz.de

† These authors contributed equally to this work.

Citation: Medina-Montano, C.; Cacicedo, M.L.; Svensson, M.; Limeres, M.J.; Zeyn, Y.; Chaves-Giraldo, J.E.; Röhrig, N.; Grabbe, S.; Gehring, S.; Bros, M. Enrichment Methods for Murine Liver Non-Parenchymal Cells Differentially Affect Their Immunophenotype and Responsiveness towards Stimulation. *Int. J. Mol. Sci.* **2022**, *23*, 6543. https://doi.org/10.3390/ijms23126543

Academic Editor: Jin-Kyu Park

Received: 20 May 2022
Accepted: 9 June 2022
Published: 11 June 2022

Publisher's Note: MDPI stays neutral with regard to jurisdictional claims in published maps and institutional affiliations.

Copyright: © 2022 by the authors. Licensee MDPI, Basel, Switzerland. This article is an open access article distributed under the terms and conditions of the Creative Commons Attribution (CC BY) license (https://creativecommons.org/licenses/by/4.0/).

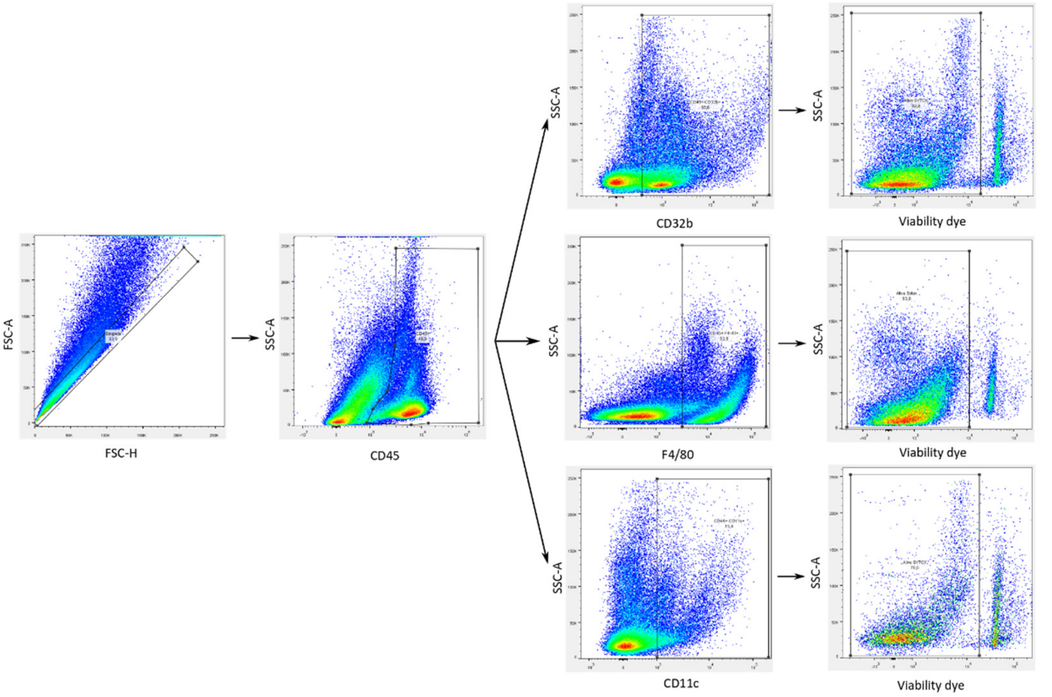

Figure S1. Gating strategy of liver NPC. After gating out debris and cell aggregates, CD45⁺ liver NPC were further identified by expression of CD32b (liver sinusoidal endothelial cells), F4/80 (macrophages) and CD11c (dendritic cells). Then, for each NPC population the frequency of viability dye negative cells was delineated (LSRII flow cytometer; analysed with FlowJo software).

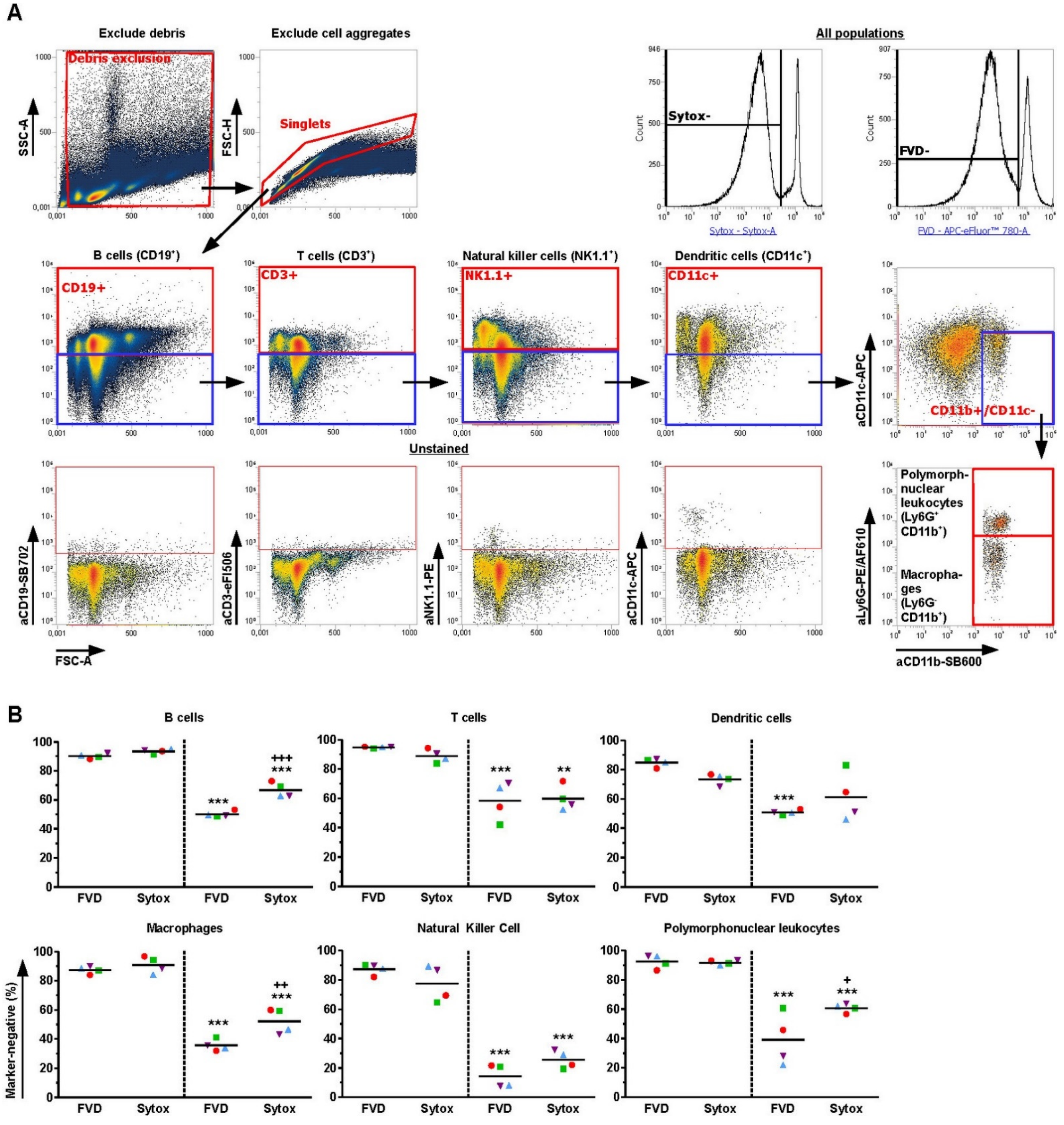

Figure S2. After overnight cultivation some splenic leukocyte populations engage FVD at higher extent than Sytox. Freshly isolated and overnight cultivated spleen cells were incubated in parallel with cell lineage-denoting antibodies, and live/dead cell-discriminating agents (FVD, Sytox). Then, samples were subjected to flow cytometric analysis. (**A**) Gating strategy. After gating out debris and cell aggregates, leukocyte populations were identified by lineage marker expression. Then, for each cell population, the frequencies of FVD and Sytox were delineated (Attune NxT Flow Cytometer; analysed with Attune NxT software). (**B**) Graphs denote the frequencies of FVD- and Sytox-negative (viable) cells (mean±SEM, n=4). Statistical significant differences versus *FVD and versus +freshly isolated are indicated (one way ANOVA, Tukey test). $^{+}p<0.05$, $^{++}p<0.01$, $^{***,+++}p<0.001$.

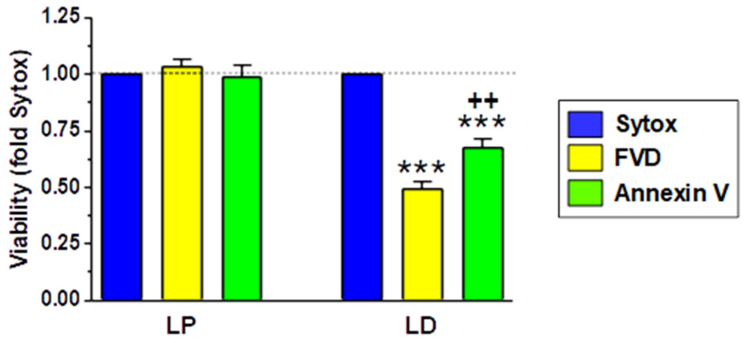

Figure S3. LD-derived liver NPC cultivated overnight engage AnnexinV at higher extent. Binding of LP- and LD-enriched CD45+ liver NPCs after overnight cultivation to various live/dead-discriminating agents was assessed by flow cytometric analysis. Graph denote the frequencies of CD45+ cells negative for Sytox-, FVD- and Annexin V (mean±SEM, n=3), normalized to values for Sytox in each experiment. Statistical significant differences versus *FVD and versus +freshly isolated are indicated (one way ANOVA, Tukey test). ++p<0.01, ***p<0.001.

Table S1. Antibodies for flow cytometry

Target	Clone	Fluorochrome
	**Liver NPC**	
CD11c	N418	PE-Cy7
CD32b	AT130-2	PE
CD45	30-F11	BV711
F4/80	BM8	eFl450
	**Spleen cells**	
CD3	145-2C11	eFl506
CD11b	M1/70	SB600
CD11c	N418	APC
CD19	1D3	SB702
NK1.1	PK136	PE
Ly6G	1A8-Ly6g	PE-eFl610
